# CLUB-MARTINI: Selecting Favourable Interactions amongst Available Candidates, a Coarse-Grained Simulation Approach to Scoring Docking Decoys

**DOI:** 10.1371/journal.pone.0155251

**Published:** 2016-05-11

**Authors:** Qingzhen Hou, Marc F. Lensink, Jaap Heringa, K. Anton Feenstra

**Affiliations:** 1 Center for Integrative Bioinformatics VU (IBIVU), VU University Amsterdam, De Boelelaan 1081A, 1081 HV Amsterdam, The Netherlands; 2 University Lille, CNRS, UMR8576 UGSF - Institute for Structural and Functional Glycobiology, F-59000, Lille, France; University of Michigan, UNITED STATES

## Abstract

Large-scale identification of native binding orientations is crucial for understanding the role of protein-protein interactions in their biological context. Measuring binding free energy is the method of choice to estimate binding strength and reveal the relevance of particular conformations in which proteins interact. In a recent study, we successfully applied coarse-grained molecular dynamics simulations to measure binding free energy for two protein complexes with similar accuracy to full-atomistic simulation, but 500-fold less time consuming. Here, we investigate the efficacy of this approach as a scoring method to identify stable binding conformations from thousands of docking decoys produced by protein docking programs. To test our method, we first applied it to calculate binding free energies of all protein conformations in a CAPRI (Critical Assessment of PRedicted Interactions) benchmark dataset, which included over 19000 protein docking solutions for 15 benchmark targets. Based on the binding free energies, we ranked all docking solutions to select the near-native binding modes under the assumption that the native-solutions have lowest binding free energies. In our top 100 ranked structures, for the ‘easy’ targets that have many near-native conformations, we obtain a strong enrichment of acceptable or better quality structures; for the ‘hard’ targets without near-native decoys, our method is still able to retain structures which have native binding contacts. Moreover, in our top 10 selections, CLUB-MARTINI shows a comparable performance when compared with other state-of-the-art docking scoring functions. As a proof of concept, CLUB-MARTINI performs remarkably well for many targets and is able to pinpoint near-native binding modes in the top selections. To the best of our knowledge, this is the first time interaction free energy calculated from MD simulations have been used to rank docking solutions at a large scale.

## Introduction

Protein-protein Interactions (PPIs) play a central role in all cellular processes. Proteins rely on their binding capacity to construct complexes and build interaction networks to fulfill biological functions (e.g., [1], [2], [3]). Knowledge of binding affinity and native binding modes of proteins are essential to gain a thorough understanding of protein-protein interactions in relation to their function (e.g., [4], [5]). During the last decades, experimental techniques such as yeast two-hybrid, tandem affinity purification, mass spectrometry and protein micro-arrays for large scale determination of protein-protein interactions, as well as immunoblots, ELISA and gel electrophoresis for calculation of binding strength, have been developed to identify and annotate protein interaction (e.g, [6–8]). However, owing to the exceeding cost of experimental techniques, computational approaches are increasingly useful to estimate binding affinity and to assist in finding the best possible interaction candidate (e.g, [9], [10]).

Molecular docking is a low-cost and fast approach to predict binding orientations, which has been developed specifically for this purpose. (e.g., [11], [5]). Docking includes two steps to predict binding modes: the first step is searching the conformational space of the protein molecules and generating (very many) docking poses. The second step is scoring the solutions constructed in the first step, with the aim of finding native or near-native ones. Often, the two steps are combined in one docking tool. Several types of algorithms exist to address the docking search problem (see Huang [12] for a recent comprehensive review). Also for the second step, various kinds of scoring functions have been developed [4][13], including, empirical or knowledge-based potentials [14][15][16] and physics-based potentials [17][18].

Although much effort has been devoted to the application of docking to PPI, accurate prediction remains a challenge that is far from solved, especially the second step which scores and ranks docking conformations. Kastritis and Bonvin (2010) found a poor correlation between scores produced by docking scoring functions and experimental binding affinities; none of the scoring function were able to predict binding affinities correctly in all situations [9]. On the other hand, it is reported that binding free energy calculation based on molecular dynamics (MD) simulations are much more accurate than docking scoring functions when used to measure binding affinities, and can even approach accuracies of experimental determination [9, 19]. One may assume that MD simulations, given long enough simulation time and an accurate force field, will yield the bound state of protein complex as its free energy minimum (e.g., [20]). However, simulations require long timescales for sufficient sampling to search the lowest free energy stable state, and are computationally very costly. To speed up these MD simulations, in our recent study [21] we successfully applied the coarse-grained (CG) MARTINI protein force field instead of a full atomic description to estimate binding affinities for proteins. Basically, we sacrifice forcefield detail for enhanced sampling. However, the CG method was shown to be as accurate as full-atom MD simulation for binding free energy estimation for the two protein complexes studied, but 500-fold less time-consuming. The achieved speedup now opens up the possibility to combine the docking searching strategy to produce a set of near-native binding decoys, which is quick and inexpensive, with estimated binding free energies from coarse-grained MD simulations, as a relatively expensive but accurate scoring method. We find this approach to achieve a good balance between efficiency and effectiveness for protein binding orientation prediction.

To illustrate this, we here evaluate the capacity of estimated binding free energy from coarse-grained MD simulations to locate near-native binding poses amongst conformations produced by docking programs. We present a proof of concept for the approach of using Free Energy calculation to score binding orientations from protein-protein docking. The benchmark dataset we used is the CAPRI Score_set, which contains over 19,000 structures from 15 published targets from the CAPRI docking experiment [13]. For most of the targets with near-native conformations we obtain significant enrichment of acceptable or higher quality structures in our top 100 selected structures. For targets with few near-native binding modes, our method can identify structures which include native interface contacts.

## Materials and Methods

### 2.1 Force fields and Software

To calculate the coarse-grained (CG) potential of mean force (PMF) for our proteins of interest, we used the procedure developed in our previous work [21]. In short, we used the MARTINI force field [22] with default time step of 20fs to perform the CG simulations. The Coarse-graining of protein structures was done using the MARTINI model which maps about 4 heavy atoms into a single interaction center. All 20 amino acids are considered as four different types of particles: polar (P), nonpolar (N), apolar (C), or charged (Q) [23]. GROMACS 4.0.5 [24] was employed to perform all MD simulations, using parameters for the MD simulations as described previously [21]. A tool that calculates the PMF from a single starting conformation in a PDB file was developed in Python. The code is available upon request.

### 2.2 Estimating Interaction strength

To evaluate the Free energy difference between bound and unbound states, one needs to integrate a path between these states. As described previously [21], a series of closely spaced distances (r) between the two centers of mass (COM) was used and for each distance a constraint force was applied to maintain the interaction of the two proteins at the set distance during the MD simulations. In detail, first, the distance between the two centers of mass of the docking conformation was calculated (dock_*COM*_). When performing the simulation, 23 distances spaced at 0.05 nm starting -0.1 nm below dock_*COM*_ and 9 distances spaced at 0.1 nm starting at 1.15 nm beyond dock_*COM*_, were used as MD simulation coordinates. From the resulting profile of force as function of distance, now the PMF was calculated as described previously [21]. The PMF describes the interaction free energy between two structures as function of distance. For each conformation of two interacting proteins, the binding free energy (△G^*off*^) was obtained from the difference between the lowest PMF value at r_*min*_ and highest PMF at larger distance r_*max*_, where r_*min*_ < r_*max*_. This binding free energy is used as an approximation of the binding strength, and in this work to rank the docking poses.

### 2.3 Datasets

To assess the ability of our method to distinguish near-native from incorrect binding modes, we used the CAPRI Score_set as a benchmark dataset [13]. CAPRI is well known as a community-wide experiment on the critical assessment of prediction of interactions. The CAPRI Score_set, which comprises around 19,000 complexed structures from multiple rounds of CAPRI experiments, is published as a realistic benchmark dataset for protein docking scoring functions. The interest of this dataset is that it does not contain the true target (crystal) structures, but includes many ‘real life’ non-occuring or incorrect, acceptable and native-like docking poses. The dataset collects structures from 15 published CAPRI targets predicted by 47 different predictor groups, which represent the state of the art of protein docking [13]. The CAPRI benchmark set used in this study is annotated regarding structural correctness, and as such does not reflect a typical research situation. However, an individual researcher may generate an input decoy dataset of any size using various docking servers that are readily available online. In this dataset, about 10% of the structures represent acceptable quality or better, based on the three measures used in CAPRI:

- *f*_*nat*_: fraction of native ligand-receptor contacts;
- L-rms: Root mean square deviation (RMSD) over the ligand backbone atoms with both of the receptors fitted on each other (docking versus solved structures);
- I-rms: RMSD of backbone atoms of interface residues between docking structures and the solved structures.

Using these measures, each structure is assigned to one of four categories based on the classification used in CAPRI:

1. High quality: *f*_*nat*_ ≥ 0.5 and L-rms ≤ 1.0 and I-rms ≤ 1.0
2. Medium quality: *f*_*nat*_ ≥ 0.5 and L-rms > 1.0 and I-rms > 1.0 or 0.3 ≤ *f*_*nat*_ < 0.5 and (L-rms ≤ 5.0 or I-rms ≤ 2.0)
3. Acceptable: *f*_*nat*_ ≥ 0.3 and L-rms > 5.0 and I-rms > 2.0 or 0.1 ≤ *f*nat < 0.3 and (L-rms ≤ 10.0 or I-rms ≤ 4.0)
4. Incorrect: *f*_*nat*_ < 0.1 or L-rms > 10.0 and I-rms > 4.0

As a measure for target difficulty, we take the fraction of docked structures of acceptable or better quality. In most figures, we organised the targets from ‘easy’ to ‘hard’ based on this measure.

### 2.4 Sampling procedure

First, binding free energy (△G^*off*^) of each structure is calculated from a single MD simulation (replicate) at each distance (in May et al., 2014 [21], 10 or 20 replicates per distance were used). Based on these free energies, we then rank the binding orientations for each target under the assumption that a lower free energy is more stable. Using this ranking, we selected the best (lowest) 50% structures for each target. For this subset, we ran four more replicates per distance to increase sampling and obtain a more accurate estimate of △G^*off*^. Based on the average △G^*off*^ over the five replicates (for the best 50% structures), we picked the top 1, 5, 10, 20 and 100 lowest binding free energies for each target. These sets we analysed and compared to the benchmark dataset. Note that, the crystal structure (PDBID: 2W83) of Target 37 includes a JNK-interacting protein JIP4 which contains a leucine zipper domain (90 Å elongated long coiled-coil) [25]. Due to its size, and the fact that simulations are performed in explicit water, this PMF calculation experiment required an excessive amount of CPU hours. Therefore, for Target 37, only one replicate simulation was performed.

To evaluate the performance of our method and compare binding strength among different quality binding poses, we also calculated the binding free energies of the X-ray crystal structures from the PDB for all targets by Coarse-Grained MD simulation using 20 replicates.

## Results and Discussion

For each of the 19,000 structures (docked conformations) of the 15 targets in the CAPRI Score_set, we estimated the binding affinity (△G^*off*^) using the CLUB-MARTINI CG MD simulations and constraint force integration, as detailed above in Methods. We then use these data to rank the docking conformations on increasing △G^*off*^ (lowest, strongest binding, first).

### 3.1 Near-native structures with lower binding free energies

**Fig 1:**
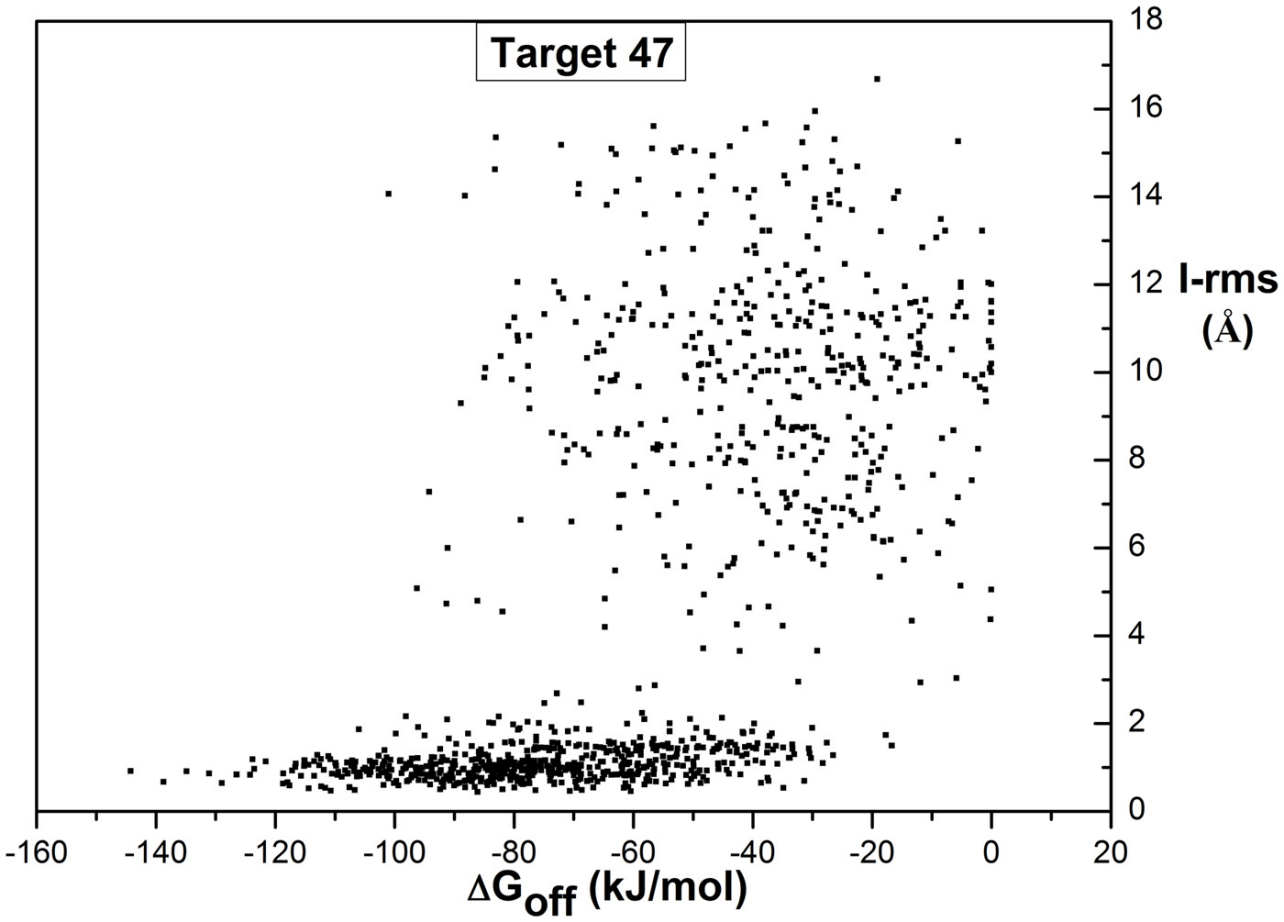
Distribution of △G^*off*^ over all structures vs. interface quality parameter I_rms (Target 47). Each dot represents one structure in the Score_set of Target 47. The x axis shows the RMSD of backbone atoms of interface residues between docking decoys and crystal structures (I_rms); the y axis shows △G^*off*^ which describes the predicted binding strength. Same plots for other targets are in S1 Fig.

To investigate the potential of △G^*off*^ as a scoring function, we first correlate the binding free energies to the Score_set benchmark parameter interface RMSD (I_rms), which is the most important determinant of the binding interface quality [13]. Fig 1 shows the comparison between △G^*off*^ (one replicate) and I-rms for all docking decoys of Target 47 (which is an easy target with many high quality docking solutions; results for other targets can be found in S1 Fig). For all docked structures, lower I_rms values reflect near-native binding poses and a lower △G^*off*^ reflects a more stable binding pose with a higher binding strength. As can be seen from Fig 1, there is a clear separation between low and high I-rms especially for lower binding free energies (△G^*off*^ < -105). Previously we showed that our method can be used to approximate experimental binding free energies [21]. The separation shown in Fig 1 demonstrates that binding free energies calculated are also accurate enough to be used as a scoring function for ranking docked conformations.

Fig 1 also shows that there is a large △G^*off*^ variation among low I_rms structures. This might reflect true differences in stability, even for very similar structures, however, we expect this to be mainly the uncertainty in our calculated △G^*off*^ which could be a limiting factor when applying our method to rank docking binding orientations. The △G^*off*^ calculations might be not accurate enough. To obtain more accurate binding free energies, more simulations are needed as already observed by May et al, 2014 [21]. To obtain some estimate of this accuracy, we selected the first target, Target 29, to run two replicate simulations for each structure in the Score_set and compare the rank position differences between the two replicates (data not shown). The average rank difference over all 2083 complexes for Target 29 was 400, which is around 25% of the number of structures for this target. Based on this variation estimate, we may select the best scoring (lowest) half on the △G^*off*^ using a single replicate simulation with reasonable reliability. For that selected best 50% of docking conformations, we ran four additional replicates to get more accurate △G^*off*^ estimates. This approach was applied to all targets. We average the △G^*off*^ calculations over the total of five replicates, and based on this we select the candidate near-native structures.

**Fig 2:**
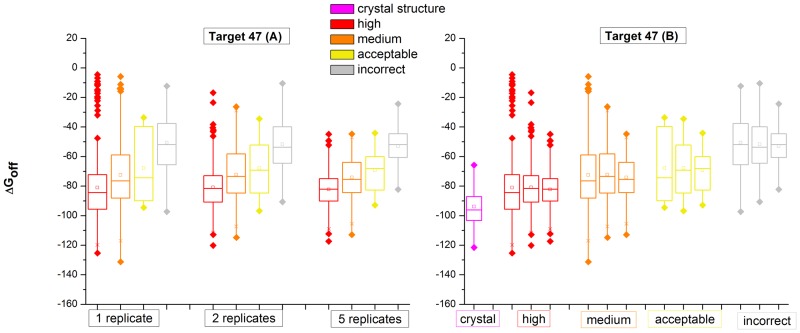
Distribution of △G^*off*^ for structures in different quality categories (Target 47). (A) △G^*off*^ distributions using 1, 2 or 5 replicate MD simulations (left to right). Each includes four bars for high, medium, acceptable and incorrect structures, from left to right, respectively. (B) Direct comparison within each quality category, and crystal structure. Each quality category contains three bars, showing the △G^*off*^ distribution using 1, 2 and 5 replicates, from left to right, respectively. Corresponding plots for the other Targets can be found in S2 Fig.

To assess whether our sampling has been sufficient to improve accurate estimations of the binding strength, we checked the distribution of △G^*off*^ in different quality categories (high, medium, acceptable and incorrect) using only 1 or 2, or all 5 replicates. For comparison, we also calculated the △G^*off*^ based on the native binding mode of the crystal structure using 20 replicates. Here we take Target 47 as an example (results for all targets are included in S2 Fig). Fig 2A shows the distributions of △G^*off*^ from 1, 2 and 5 replicate MD simulations. The four bars for each represent high (red), medium (orange), acceptable (yellow) and incorrect (gray) structures respectively. From the plot, we observe that the mean △G^*off*^ separates clearly between high, medium, acceptable and incorrect. Moreover, as hypothesized, the predicted binding free energy increases from high to low quality structures. This clearly shows that CLUB-MARTINI is able to predict lower binding free energies for near-native binding poses. Fig 2B shows that the spread of △G^*off*^ within each quality category decreases with increasing number of replicates. Moreover, for ‘high’ quality structures, the mean △G^*off*^ is quite stable and close to that of the crystal structures (in purple) and gets even closer with more replicates. This trend suggests that further improvement of binding free energy calculation may be possible with more replicates. For the other targets, we observe similar trends in most cases (see S2 Fig). For Target 50 (S2 Fig), we see that the initial (single replicate) △G^*off*^ for high quality structures is **higher** than that of the crystal structure, which is likely due to errors arising from using a limited number of replicates. Reassuringly, we indeed see this gap decreasing when more replicates are used.

### 3.2 Enrichment in selecting near-native structures in CAPRI Score set

**Fig 3:**
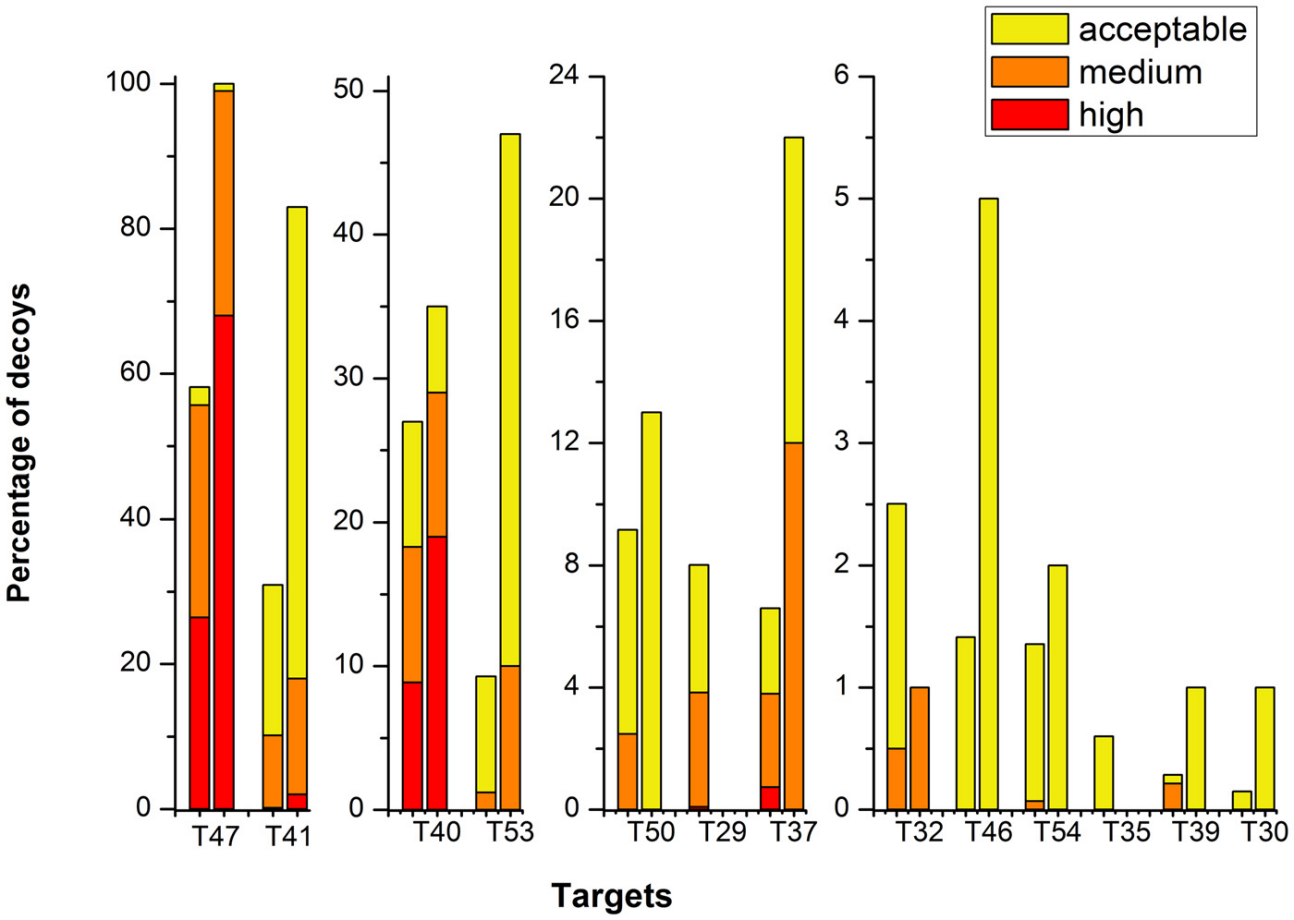
Enrichment of acceptable or better structures. For each of the 13 targets with acceptable or better decoys, two columns (from left to right) show the CAPRI Score_set and the top 100 in our rank of binding free energy calculation. Red, orange and yellow represent the fractions of high, medium and acceptable quality structures, respectively, of the number of the selected docking decoys. The ordering of the targets is based on the fraction of acceptable or better structures in each target; easy targets with a high fraction are on the left, hard targets with low fraction on the right. Note that for improved readability, the vertical scale decreases from left to right.

After ranking our docking structures based on the binding free energy calculation, we compared the percentage of acceptable or better quality structures in our top selections with that in CAPRI Score_set. Fig 3 shows the comparison of the top 100 structures for each target. The two bars represent the percentage of structures in each quality category in Score_set and our top 100 selections, respectively. The targets in Fig 3 are ordered on the fraction of acceptable or better structures in the CAPRI Score_set, which should correspond to the level of difficulty in docking prediction [26][27] (note that for clarity the y axis scale decreases from left to right). For almost all of the Targets that include near-native binding orientations in the Score_set, CLUB-MARTINI can retain at least acceptable quality structures in the top 100 selection, with two exceptions, target 29 and target 35, which will be discussed below in section 3.5 Future Improvements. Moreover, for most of the targets, our method enriches the percentage of near-native structures (high in red, medium in orange and acceptable in yellow in Fig 3), except for targets 29, 32 and 35. For target 47, which is an easy docking problem, our complete top 100 selection is of at least acceptable and by far most are of high quality. In addition, for targets which have high quality models in the data set (T47, T41, T40, T29), our approach always enriches that fraction, with the single exception of target 29 which has only 2 high quality conformations in 2083 structures to begin with. It turned out that the small random variations in the single replicate △G^*off*^ caused these to be lost during the first ranking. From these results, we can conclude that CLUB-MARTINI is able to enrich near-native binding modes at any difficulty level.

**Fig 4:**
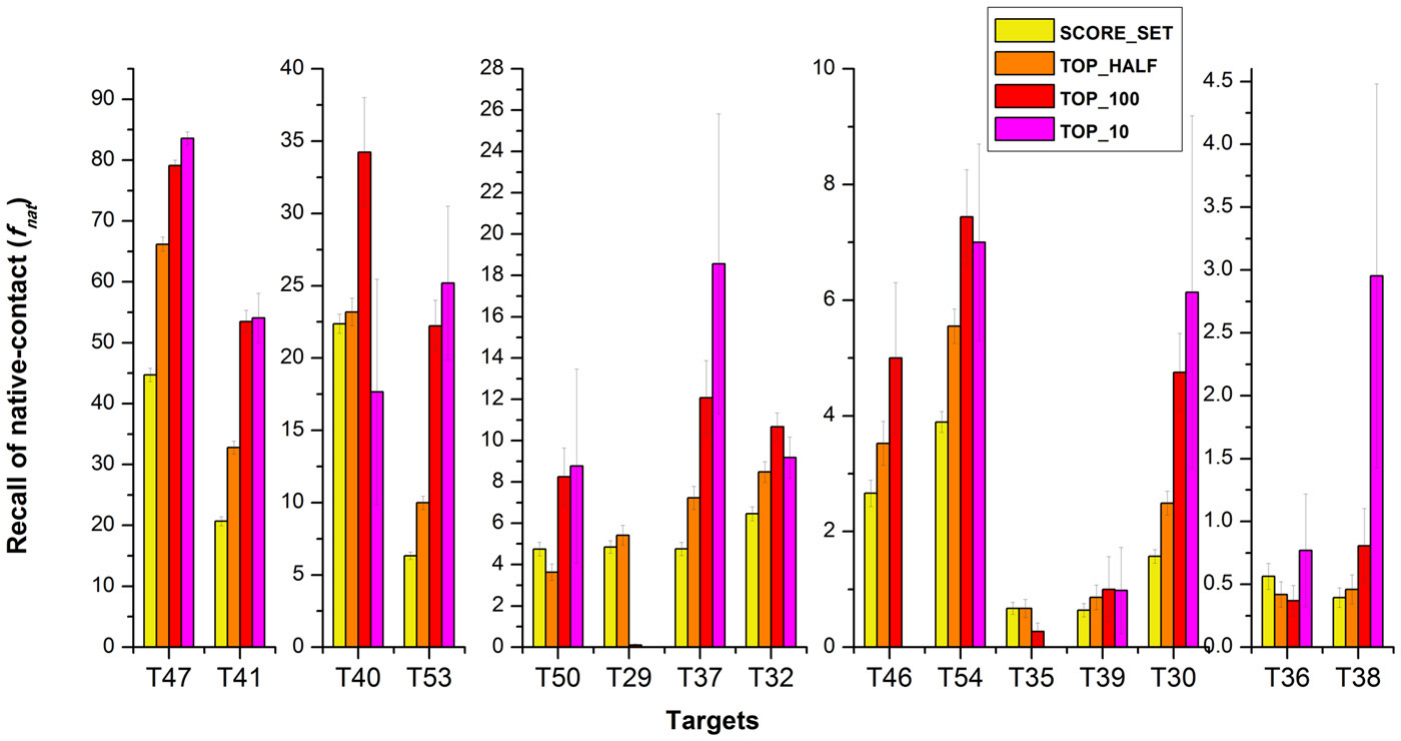
Enrichment of native-contacts between Score_set and our method. Four bars for each target represent the Recall in Score_set, Top_half, Top_100 and Top_10, respectively (left to right). For most of the Targets, Top_10 obtains the best Recall. The ordering of the targets is the same as in Fig 3. (hardest on the right). Note that for improved readability, the vertical scale decreases from left to right.

For Target 36 and Target 38 (the hardest docking problems), there are no acceptable conformations in the data set, i.e., they are all ‘incorrect’. To also illustrate our prediction performance here, we compare our method based on the native interface contacts using average Recall (*f*_*nat*_) and Precision (1-*f*_*nonnat*_) as defined in Score_set [13]. This measure is similar to using the fraction of predicted binding residues [28], but the fraction of native contacts is more sensitive to the interface quality. Fig 4 shows the comparison among Score_set, our top_half, top_100 and top_10 for all targets. The fact that the Recall increases from Score_set, top_half, to top_100 for most targets shows that our method is able to enrich the interface native-contact. For the two most difficult targets (T36, T38), containing only incorrect models, we do obtain structures with native binding contacts, and in particular we obtain a significant enrichment for Target 38 (Precision follows the same trend as Recall, as can be seen in S3 Fig).

### 3.3 Selecting near-native structures in our top ranked solutions

Table 1 summarizes the quality of structures selected in the Top 1, 5, 10 and 20 for multiple targets. For the top 1, we can select one high quality structure for Target 47, and one acceptable for Target 53 (2 targets out of 13). In the Top 5, we can select acceptable or better ones for two additional targets (4/13) and again one more (5/13) in the Top 10 and six targets (6/13) in the Top 20. This indicates that our method is quite able to enrich native binding modes for almost half the targets. CLUB-MARTINI is indeed capable of obtaining favourable candidates within a reasonably small selection, which shows its potential to be directly used to limit the range of putatively correct binding solutions that may aid other expensive experiments, such as for example in drug design. Furthermore, such a selection of candidates is small enough to be subjected to visual inspection, which may further help to pinpoint the solutions of highest relevance. In practice, when investigating how two proteins interact with each other, we envision an approach in which one first generates a set of docking solutions using several cheap docking approaches, and then our CLUB-MARTINI can be employed to rank the docking orientations and find the most relevant binding poses.

**Table 1:** Success selections of top ranked structures.

Selection	Target	Quality			Total (%)
		High	Medium	Acceptable	
TOP 1	T47	1	0	0	100
	T53	0	0	1	100
TOP 5	T47	3	2	0	100
	T41	0	0	4	80
	T53	0	0	3	60
	T37	0	2	0	40
TOP 10	T47	7	3	0	100
	T41	0	1	7	80
	T53	0	1	5	60
	T37	0	3	0	30
	T50	0	0	1	10
TOP 20	T47	14	6	0	100
	T41	0	4	13	85
	T53	0	3	9	60
	T37	0	4	2	30
	T50	0	0	3	15
	T40	1	2	0	15

The table is derived from 5-replicate MD simulation for all targets, except Target 37 where only one replicate simulation was available due to limits on computational resources.

### 3.4 Comparison between CLUB-MARTINI and other scoring methods in CAPRI

We also compare our method with the overall performance of other scorers participating in the CAPRI scoring rounds [29], [27, 30]. Each ‘scorer’ ranked and submitted up to 10 selections from an anonymized set of docked conformations uploaded by ‘predictors’. The Score_set in this paper is identical to an anonymized set, with the exception of a few structures removed in the construction of Score_set based on additional tests (for example, minimum sequence coverage of 70%)[13]. Note that, ‘scorers’ are allowed to modify the selected structures, so sometimes what scorers submit is of better quality than what they initially selected. In the current work, we did not attempt such improvements, although the simulation based method on principle could support that.

**Fig 5:**
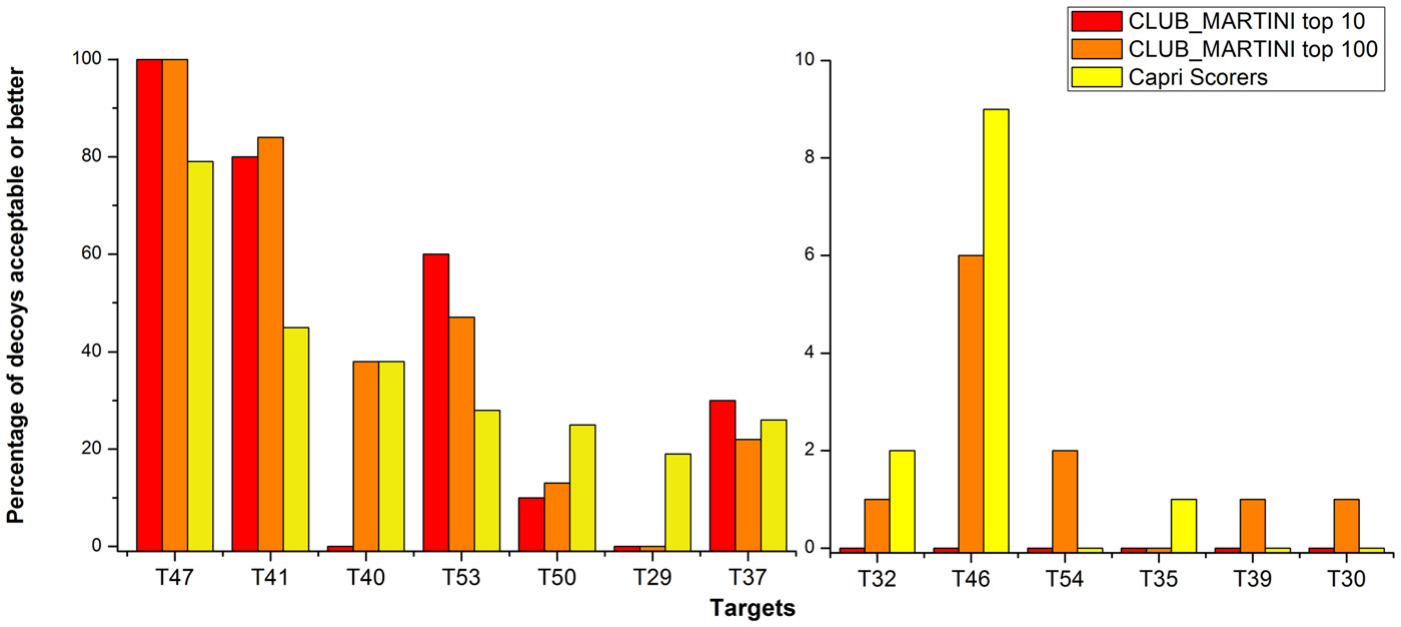
Comparison with overall performance of ‘scorers’ in CAPRI experiments. There are three bars for each target: red for CLUB-MARTINI Top_10, orange for CLUB-MARTINI Top_100 and yellow for CAPRI ‘Scorers’. We consider our Top_100 performance significantly better for five targets: T41, T53, T54, T39 and T30 (higher than 1.5 fold relative to CAPRI Scorers); and similar for four more: T47, T40, T37 and T46 (0.67 to 1.5 fold). The ordering of the targets is the same as in Fig 3 (hardest on the right).

Fig 5 shows the comparison between the percentage of acceptable or better models in our top 10 and top 100 selections and the overall selection of ‘scorers’ in CAPRI. Our top 100 is significantly better for five targets: T41, T53, T54, T39, T30 (5/13) and similar for four more: T47, T40, T37, T46 (in total 9/13 better or similar). Our top 10 selections obtain similar or higher fractions acceptable or better for T47, T41, T53, T37 (4 out of 13 targets).

In addition to this overall comparison between our method with the average ‘scorer’ performance in CAPRI, we also compared our method with the performance of individual state-of-the-art ‘scorers’ in the CAPRI rounds. Table 2 shows details of the near-native binding poses identified by seven best ‘scorers’. Targets without near-native solutions and those for which all the seven ‘scorers’ did not predict, are not shown in the table. Full details for all targets and scorers are presented in S1 Table. For most targets for which these ‘best scorers’ give at least acceptable quality predictions, our method is also able to select near-native binding modes (5/9). Moreover, we rank 2nd for Target 53, 3rd for Target 47, 3rd for Target 41, 5th for Target 37 and 7th for Target 50. Our method is not able to find the correct binding modes in our top 10 selections for two ‘hard’ targets (Target 46 and Target 35) and two ‘easy’ targets (Target 29 and Target 40). We will discuss some possible reasons for this in the next section. In conclusion, our method works well for most targets and even better than the majority of the ‘best’ scoring functions for some targets.

**Table 2:** Comparison of top 10 selections between CLUB_MARTINI and state of art docking scoring functions.

Groups	T47	T46	T50	T53	T29	T35	T37	T40	T41	Total
Bates	10/10**	2*	2*	1/1**	4/2**		6/1***	10/9**	4*	8
Bonvin	10/9***/1**	2*	2*	8/3**	9/5**		2/1**	10/2***	10*	8
Elber		1*	2*	5/1**				8/3***	1*	5
Fernandez-Recio	10/4***/6**		6/1**	4/1**	5/1***	0			3/2**	5
Wang	2/2***		7/6**	5/1**		1*	6/4**	8/1***	7*	7
Weng	9/6***/3**	3*	1*	3/1**	3/2**		2/1***	7/2***	4*	8
Zou	10/10***	1*	2/1**	1*			4/2***	10/2***	10/2***	7
**CLUB_MARTINI**	10/7***/3**	0	1*	6/1**	0	0	3**	0	8/1**	5

The Table lists the number of acceptable or better quality models from different groups. For each group, the number of submitted correct models of each category are listed: high(***), medium(**), and acceptable (*) are listed. For example, ‘10/7***/3**’ means that there are 10 acceptable or better quality binding poses in total, of which 7 structures are with high quality and 3 models are with medium accuracy. Note that all number in the table except our method are from previous CAPRI paper [26][27].

### 3.5 Further Improvement

As shown above, CLUB-MARTINI can enrich near-native structures for most targets, even for ‘hard’ ones with no or few high or medium quality decoys. Even for Target 37, we can obtain strong enrichment even though we only performed a single replicate simulation due to computational constraints. Nevertheless, our method is unable to obtain high performance for some targets, notably, Target 29 and Target 35, as reflected by both the enrichment and precision analysis. Here we will discuss same contributing factors to this low performance for these two targets. S2 Fig shows that for T29 the average binding free energies of ‘incorrect’ structures are lower than those of the crystal structure. This explains why our method based on binding free energies calculation was unable to separate different quality binding poses. There may be several reasons for this. First, the CG MARTINI forcefield may not accurately represent the interaction for this particular pair of proteins. Second, the quality of the docking conformations for each target is judged based on the respective crystal structure. However, the crystal structure may not reflect the functionally relevant binding conformation; or there may be alternate binding modes of which the crystal structure only represents single one, while the forcefield captures another. For example, in Fig 1, some incorrect structures (high I-rms ones) have low binding free energies; some of these might represent alternative binding modes, and hence have favourable free energies. This factor may introduce errors into the evaluation of our ranking based on binding free energy calculations. For Target 40, a special situation occurs, that the crystal structure contains two distinct binding modes. In S2 Fig, we show the binding free energy for both of them, and we can observe that one has a significantly lower binding free energy than the other. When ranking decoys for such a target, only binding modes corresponding to the lowest will end up being selected. Without concrete data on alternative binding modes from a crystal structure, we cannot know if this may be the case as well for other targets.

In this work, we spent 1.1 million CPU hours for all simulations. Although we speed up MD simulation by using the coarse-grained model, the computational cost clearly is still a limiting factor for our method. By clustering conformations of similar orientation, either from the docking or the snapshots from the simulations, we may obtain effectively increased sampling and save on CPU hours for that particular binding orientation.

We investigated whether better enrichment may be achieved when more replicate simulations are performed. S4 Fig shows the percentage of acceptable or better structures in our top 100 selection for all targets using 1, 2 or 5 replicates. Three bars for each target represent the enrichment for 1, 2 and 5 replicates from left to right. For most targets, despite the increased accuracy of △G^*off*^ as shown above (Fig 2 & S2 Fig), there are no big differences in enrichment with more replicates, except for T40 and T50 whose enrichment increases more than 5 percent points. As shown in Fig 2 and S2 Fig, the variance of binding free energy for near-native structures shrinks with more replicates and gets closer to the ‘real’ structures. This indicates the binding free energies become more accurate with increased sampling. For targets where the binding free energy of the crystal structure is unfortunately higher than the incorrect binding poses, for example target 29, more replicates would not lead to improved enrichment. However, for most of the targets the interaction free energy of the crystal structure is lower than that of the incorrect structures. Therefore, we would expect that using more replicates (e.g., 10 or 20 as in our original analysis [21]) would further improve the enrichment. This opens up the possibility for researchers to attain higher precision by investing more CPU resources for selected targets.

## Conclusions

Ranking all docking solutions effectively and identifying the native-solutions correctly are the leading demand of a scoring function. Here, we have shown that calculating binding free energies from Coarse-grained MD simulations using the MARTINI force-field can be an effective way to detect favourable interactions amongst many diverse binding orientations. To the best of our knowledge, this is the first time that interaction free energy from force field simulations is used as a scoring method to rank docking solutions at a large scale. As an outlook, our approach, unlike most other docking scoring functions, is closer to the physical reality, which opens up the possibility to provide an absolute and quantitative description of protein interactions, allowing the prediction of complete interactomes in *silico*.

## Supporting Information

S1 Fig Distribution of △G^*off*^ over all structures vs. interface quality parameter I-rms. Each dot represents one structure in the Score_set of Targets. The x axis shows the RMSD of backbone atoms of interface residues between docking decoys and the crystal structure; the y axis represents the △G^*off*^ which describes the binding strength.
(PDF)

S2 Fig Distribution of △G^*off*^ for structures in different quality categories for all Targets. (A) Distributions of △G^*off*^ from 1, 2 or 5 replicate MD simulations (left to right). Each set of △G^*off*^ includes four bars which stand for high, medium, acceptable and incorrect structures respectively (left to right). (B) Direct comparison of the same quality category for △G^*off*^ from 1, 2 or 5 replicates. Each quality category contains three bars: distribution from 1, 2 and 5 replicates (left to right). Note that Target 37 includes a JNK-interacting protein JIP4 which contains a leucine zipper domain (90 Å extended long coiled-coil)[25]. It needs huge amount of CPU-hours even with coarse-grained model and the result is based on only one replicate simulation. * Target 40 has two native interfaces (between chain AC and BC).
(PDF)

S3 Fig Comparison of precision of native contacts between Score_set and our method. Four bars for each target represent precision in Score_set, Top_half, Top_100 and Top_10, respectively (left to right).
(PDF)

S4 Fig Comparison of acceptable or better structures in Top 100 selection using one, two or five replicate simulations. Three bars (left to right) of each target represent top 100 selection in one, two and five replicate simulations. (The last bars for each targets correspond to the middle bar in Fig 3.)
(PDF)

S1 Table The performance of all docking scoring functions participating CAPRI for all 15 Targets in CAPRI Score_set. All docking scoring functions participating the CAPRI competitions for the 15 targets and the number of acceptable or better quality models selected by these methods are listed. For each group, the number of submitted correct models of each category are indicated: high accuracy (***), medium accuracy (**), and acceptable (*). Data obtained from [26] and [27].
(PDF)

S1 File The PMF calculation results for all 15 targets (S1_File.zip). (ZIP)
