# Supplementary material for: CLUB-MARTINI: Selecting Favourable Interactions amongst Available Candidates, a Coarse-Grained Simulation Approach to Scoring Docking Decoys
Source: PLoS One. 2016 May 11;11(5):e0155251. doi: 10.1371/journal.pone.0155251 (PMC4864233; doi:10.1371/journal.pone.0155251)

Fig. 1. Distribution of ΔG^{off} over all structures vs. interface quality parameter I_rms. Each dot represents one structure in the Score set of Targets. The x axis shows the RMSD of backbone atoms of interface residues between docking decoys and the crystal structure; the y axis represents the ΔG^{off} which describes the binding strength.

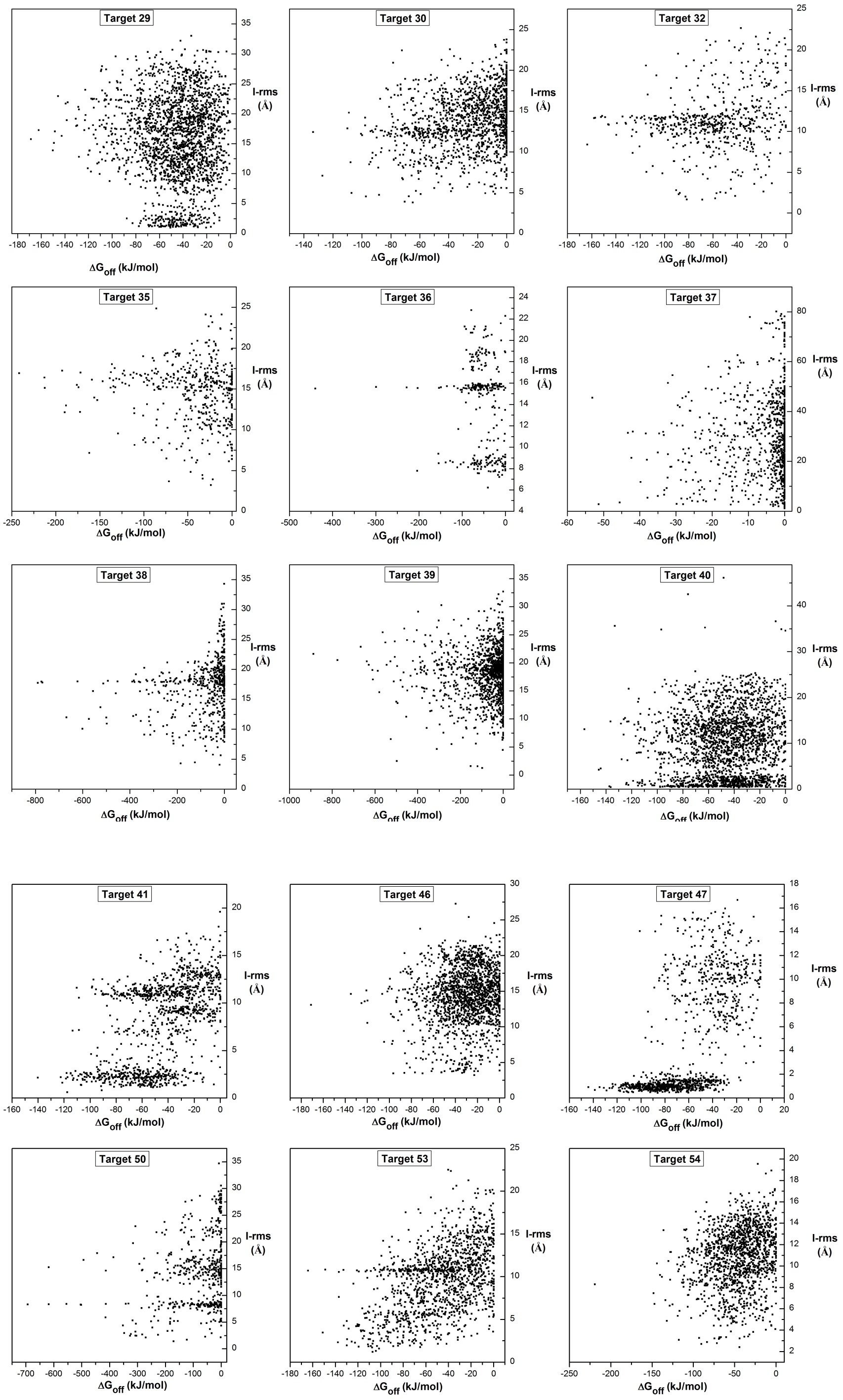

Supplement: S1 Fig — Each dot represents one structure in the Score_set of Targets. The x axis shows the RMSD of backbone atoms of interface residues between docking decoys and the crystal structure; the y axis represents the △Goff which describes the binding strength. (PDF) [file pone.0155251.s001.pdf]
