# Supplementary material for: CLUB-MARTINI: Selecting Favourable Interactions amongst Available Candidates, a Coarse-Grained Simulation Approach to Scoring Docking Decoys
Source: PLoS One. 2016 May 11;11(5):e0155251. doi: 10.1371/journal.pone.0155251 (PMC4864233; doi:10.1371/journal.pone.0155251)
Supplement: S2 Fig — (A) Distributions of △Goff from 1, 2 or 5 replicate MD simulations (left to right). Each set of △Goff includes four bars which stand for high, medium, acceptable and incorrect structures respectively (left to right). (B) Direct comparison of the same quality category for △Goff from 1, 2 or 5 replicates. Each quality category contains three bars: distribution from 1, 2 and 5 replicates (left to right). Note that Target 37 includes a JNK-interacting protein JIP4 which contains a leucine zipper domain (90 Å extended long coiled-coil)[25]. It needs huge amount of CPU-hours even with coarse-grained model and the result is based on only one replicate simulation. * Target 40 has two native interfaces (between chain AC and BC). (PDF) [file pone.0155251.s002.pdf]

Fig. 2. Distribution of ΔG^{off} for structures in different quality categories for all Targets.
(A) Distributions of ΔG^{off} from 1, 2 or 5 replicate MD simulations (left to right). Each set of ΔG^{off} includes four bars which stand for high, medium, acceptable and incorrect structures respectively (left to right). (B) Direct comparison of the same quality category for ΔG^{off} from 1, 2 or 5 replicates. Each quality category contains three bars: distribution from 1,2 and 5 replicates (left to right) . Note that Target 37 includes a JNK-interacting protein JIP4 which contain a leucine zipper domain (90 Å extended long coiled-coil) (Isabet et al., 2009). It needs huge amount of CPU-hours even with coarse-grained model and the result is only based on one replicate simulation. * Target 40 has two native interfaces (between chain AC and BC)

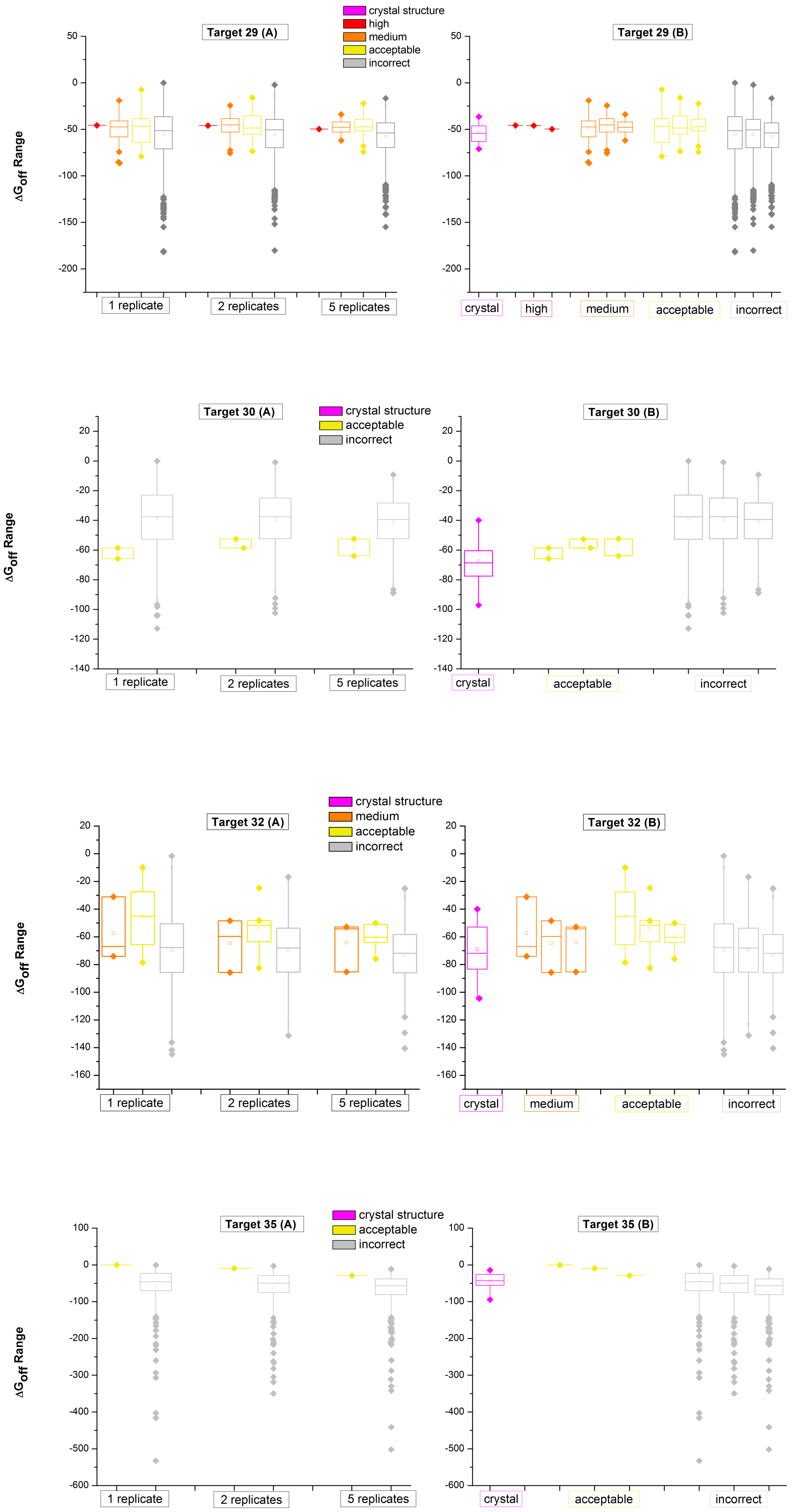

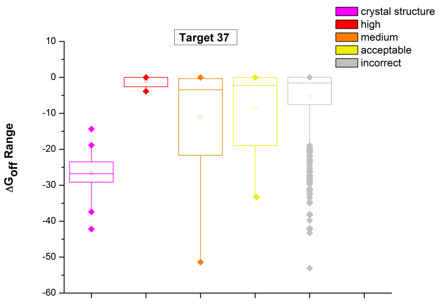
Target 37
crystal structure
high
medium
acceptable
incorrect
ΔGoff Range

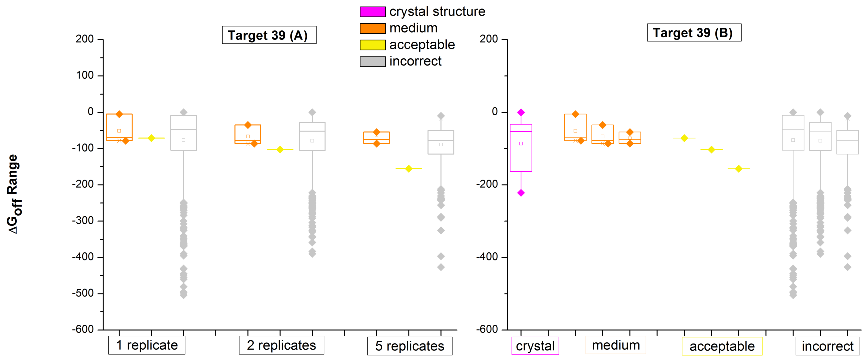
Target 39 (A)
Target 39 (B)
crystal structure
medium
acceptable
incorrect
ΔGoff Range
1 replicate
2 replicates
5 replicates
crystal
medium
acceptable
incorrect

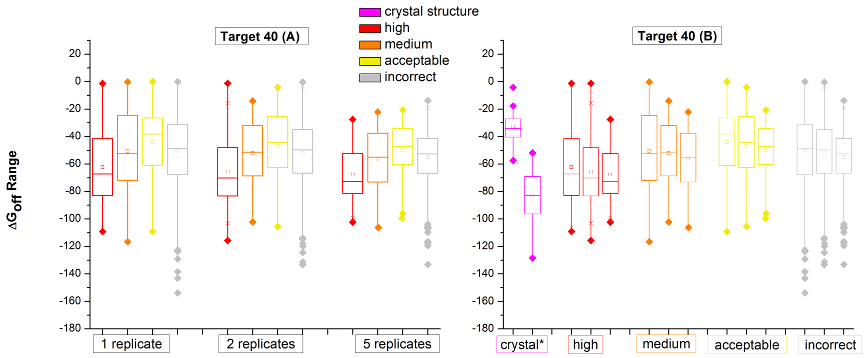
Target 40 (A)
Target 40 (B)
crystal structure
high
medium
acceptable
incorrect
ΔGoff Range
1 replicate
2 replicates
5 replicates
crystal*
high
medium
acceptable
incorrect

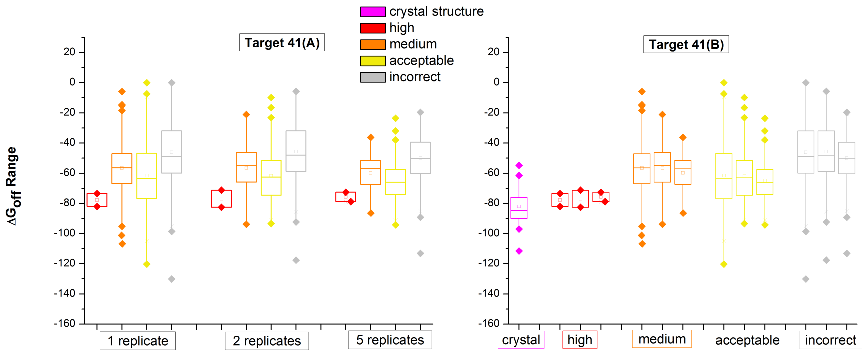
Target 41(A)
Target 41(B)
crystal structure
high
medium
acceptable
incorrect
ΔGoff Range
1 replicate
2 replicates
5 replicates
crystal
high
medium
acceptable
incorrect

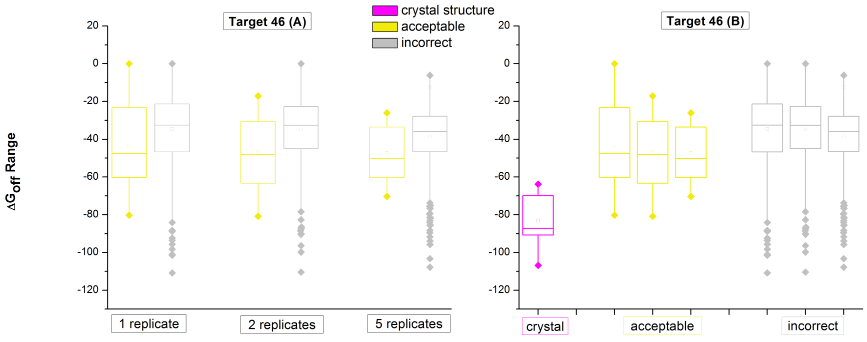
Target 46 (A)
Target 46 (B)
crystal structure
acceptable
incorrect
ΔGoff Range
1 replicate
2 replicates
5 replicates
crystal
acceptable
incorrect

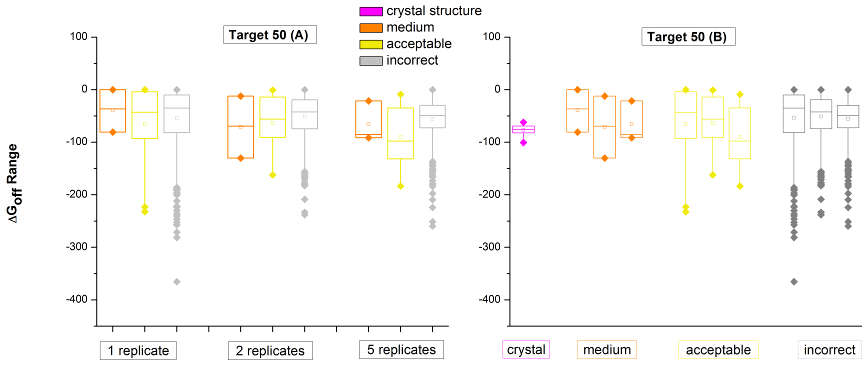
Target 50 (A)
Target 50 (B)
crystal structure
medium
acceptable
incorrect
ΔGoff Range
1 replicate
2 replicates
5 replicates
crystal
medium
acceptable
incorrect

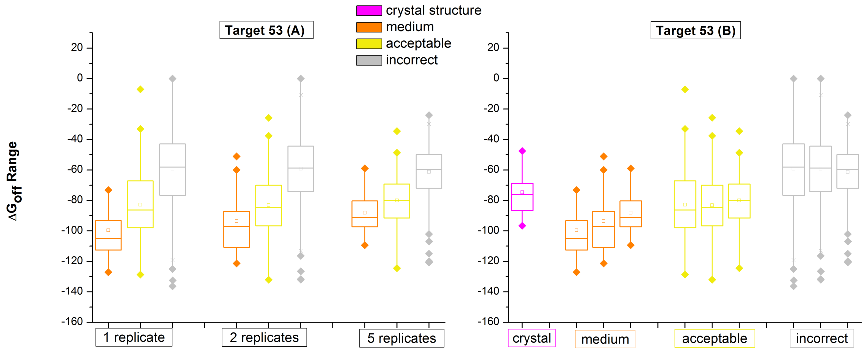
Target 53 (A)
Target 53 (B)
crystal structure
medium
acceptable
incorrect
ΔGoff Range
1 replicate
2 replicates
5 replicates
crystal
medium
acceptable
incorrect

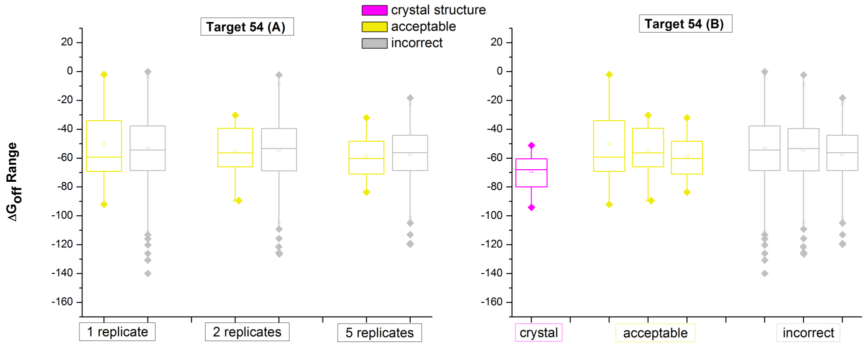
Target 54 (A)
Target 54 (B)
crystal structure
acceptable
incorrect
ΔGoff Range
1 replicate
2 replicates
5 replicates
crystal
acceptable
incorrect
