# Supplementary material for: CLUB-MARTINI: Selecting Favourable Interactions amongst Available Candidates, a Coarse-Grained Simulation Approach to Scoring Docking Decoys
Source: PLoS One. 2016 May 11;11(5):e0155251. doi: 10.1371/journal.pone.0155251 (PMC4864233; doi:10.1371/journal.pone.0155251)

Fig. 3. Comparison for precision of native-contact between Score_set and our method.
Four bars for each target represent precision in Score_set, Top_half, Top_100 and Top_10, respectively (left to right).

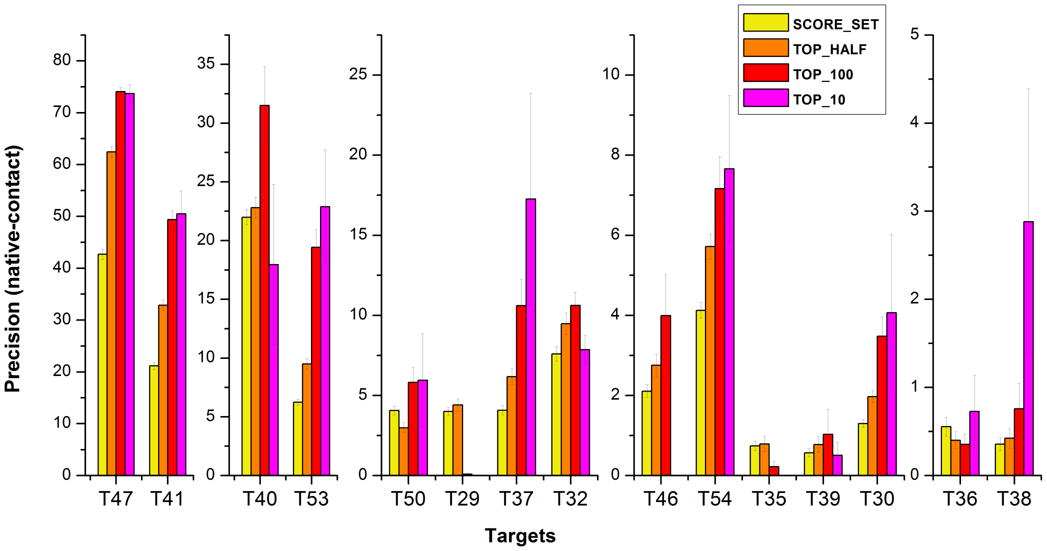

Supplement: S3 Fig — Four bars for each target represent precision in Score_set, Top_half, Top_100 and Top_10, respectively (left to right). (PDF) [file pone.0155251.s003.pdf]
