# Supplementary material for: CLUB-MARTINI: Selecting Favourable Interactions amongst Available Candidates, a Coarse-Grained Simulation Approach to Scoring Docking Decoys
Source: PLoS One. 2016 May 11;11(5):e0155251. doi: 10.1371/journal.pone.0155251 (PMC4864233; doi:10.1371/journal.pone.0155251)

Fig. 4. Comparison of acceptable or better structures in Top 100 selection of one, two or five replicate simulations.
Three bars (left to right) of each target represent top 100 selection in one, two and five replicate simulations. (The last bars for each targets correspond to the middle bar in Fig. 3.

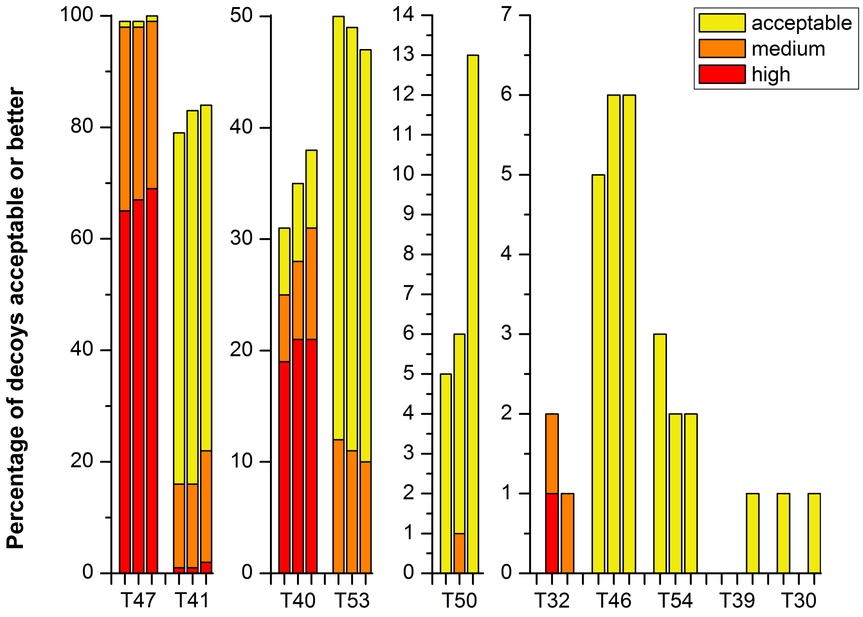

Supplement: S4 Fig — Three bars (left to right) of each target represent top 100 selection in one, two and five replicate simulations. (The last bars for each targets correspond to the middle bar in Fig 3.) (PDF) [file pone.0155251.s004.pdf]
