# Supplementary material for: CLUB-MARTINI: Selecting Favourable Interactions amongst Available Candidates, a Coarse-Grained Simulation Approach to Scoring Docking Decoys
Source: PLoS One. 2016 May 11;11(5):e0155251. doi: 10.1371/journal.pone.0155251 (PMC4864233; doi:10.1371/journal.pone.0155251)
Supplement: S1 Table — All docking scoring functions participating the CAPRI competitions for the 15 targets and the number of acceptable or better quality models selected by these methods are listed. For each group, the number of submitted correct models of each category are indicated: high accuracy (***), medium accuracy (**), and acceptable (*). Data obtained from [26] and [27]. (PDF) [file pone.0155251.s005.pdf]

Table 1. The performances of all docking scoring functions participating CAPRI for all 15 Targets in CAPRI Score set. All docking scoring functions participating the CAPRI competitions for the 15 targets and the number of acceptable or better quality models selected by these methods are listed. For each group, the number of submitted correct models of each category are indicated:high accuracy (***), medium accuracy (**), and acceptable (*). Data obtained from [26] and [27].

Group	Target 47	Target 46	Target 50	Target 53	Target 54	Target 29	Target 30	Target 32
Bates	10/10**	2	2	1/1**	0	4/2**	0	
Bonvin	10/9***/1**	2	2	8/3**	0	9/5**		
Weng	9/6***/3**	3	1	3/1**		3/2**		
Wang	2/2***		7/6**	5/1**				
Zou	10/10***	1	2/1**	1	0			
Elber	x	1	2	5/1**				
Fernandez-Reci	10/4***/6**		6/1**	4/1**		5/1***		
Xiao	x	1	6/4**	3				
Camacho	10/9***/1**					2/1**		
Gray	9/3***/6**		4/1**	1/1**				
Grudinin	5/3***/2**		1	3/1**				
Haliloglu	1/1***							
Kihara	7/6***/1**	4						
Wolfson	x	1				2*		
Korkin	10/3***/7**		1/1**					
Takeda-Shitaka							0	
Bajaj			1					
Mitchell	x							
Seok	x			2/2**				
Umeyama	8/7***/1**							
Vajda	x		x	x		2/1**		
Vakser	x		x					2*
AZE								
Liu								
Samson+Hex								
CLUSPRO	x							
Cui	x							
Eisenstein	x							
FIBERDOCK	x							
HADDOCK	x	x						
HEXSERVER	x							
Luethy	x							
Nakamura	x							
Ritchie	x							
Shen	x							
Zacharias	x						x	
Zhou	x					0		
Pal			0					
Poupon				0				
Ten Eyck						0		
Smith						0		

**Group**	**Target 35**	**Target 37**	**Target38**	**Target39**	**Target40**	**Target41**	Total
Bates		6/1***			10/9**	4*	8
Bonvin		2/1**			10/2***	10*	8
Weng		2/1***		0	7/2***	4*	8
Wang	1*	6/4**			8/1***	7*	7
Zou		4/2***			10/2***	10/2***	7
Elber					8/3***	1*	5
Fernandez-Recio				0		3/2**	5
Xiao						7/4**	4
Camacho		0			10/9***		3
Gray							3
Grudinin					0		3
Haliloglu		5/4**			6/4***		3
Kihara						3/1**	3
Wolfson		1*			9/6***		3
Korkin							2
Takeda-Shitaka					10***	10/2**	2
Bajaj							1
Mitchell		2*					1
Seok							1
Umeyama							1
Vajda							1
Vakser							1
AZE		1***					1
Liu					4/4**		1
Samson+Hex						10/1**	1
CLUSPRO							
Cui							
Eisenstein							
FIBERDOCK							
HADDOCK							
HEXSERVER							
Luethy							
Nakamura							
Ritchie							
Shen							
Zacharias							
Zhou							
Pal							
Poupon							
Ten Eyck							
